# Risk Factors for Diabetic Foot Ulcers and the Role of Thermal Imaging in Early Detection

**DOI:** 10.7759/cureus.107878

**Published:** 2026-04-28

**Authors:** Ikram Damoune, Oumaima Mandari, Hassan Douzi, Rachid Harba, Bouchra Rherissi, Hafid Elfahimi

**Affiliations:** 1 Department of Endocrinology, Mohammed VI University Hospital of Agadir, Agadir, MAR; 2 REGNE Laboratory, Faculty of Medicine, Ibn Zohr University, Agadir, MAR; 3 IRF-SIC Laboratory, Faculty of Sciences, Ibn Zohr University, Agadir, MAR; 4 Polytech Orléans, PRISME Laboratory, University of Orléans, Orléans, FRA; 5 Laboratory of Cellular Biology and Molecular Genetics, Faculty of Sciences, Ibn Zohr University, Agadir, MAR

**Keywords:** diabetic foot, hyperthermia, lower limb amputation, thermal camera, ulcer

## Abstract

Background and aim

Diabetes is the main cause of foot ulcers and limb amputation. The primary aim of our study was to identify clinical risk factors for the development of foot ulcers in patients with diabetes. The secondary aim was to evaluate the benefits of using a thermal camera and AI to detect feet at high risk of developing foot ulcers.

Methods

A two-year prospective study was conducted including all diabetic patients admitted to the endocrinology department at Mohammed VI University Hospital of Agadir. Data were collected from patient records, and thermal imaging was performed in all diabetic patients, with or without foot ulcers, after obtaining informed consent.

Results

The study included 158 diabetic patients admitted to the endocrinology department at Mohammed VI University Hospital of Agadir. Foot ulcer prevalence was 14.6% (n = 23). In patients with ulcers, the average age was 51 years, with a predominance of males (56%) (n = 13); the average time since diagnosis of diabetes was 6.5 years. High blood pressure was found in 26% (n = 6) of the patients, hyperlipidemia in 30% (n = 7), smoking in 47% (n = 11), and overweight in 34% (n = 8), with an average body mass index of 27 kg/m², poor diabetes control with an average hemoglobin A1c of 11%, and a high average fasting blood glucose level of 3.6 g/L. Foot examination of patients with ulcers revealed neuropathy in 86% (n = 20) and peripheral artery disease with a low ankle-brachial index in 82% (n = 19). We found that 56% (n = 13/23) of the patients with ulcers had a positive thermal imaging delta on admission, with an average delta of 3.3 ± 0.6 °C. In the group of patients with ulcers, a statistically significant correlation was found with smoking (p = 0.001), overweight (p = 0.017), poor glycemic control (p = 0.002), diabetic retinopathy (p < 0.001), diabetic neuropathy (p < 0.001), peripheral artery disease (p < 0.001), and foot deformity (p < 0.001). Finally, high thermal delta was found to be significantly more common in the ulcer group (p < 0.001).

Conclusions

Evaluating a foot at risk of ulceration requires time and trained medical staff, making thermal imaging cameras useful for detecting hyperthermia 15 days before an ulcer appears. The assessment of a diabetic foot at risk of developing an ulcer requires a physical examination to identify the main risk factors in our study: neuropathy, arteriopathy, and foot deformity. Hyperthermia detected by a thermal imaging camera is a new marker that may enable the early detection of foot ulcers, allowing earlier intervention to prevent amputations.

## Introduction

Diabetes is recognized as a true global pandemic. The severity of diabetes is due to micro- and macroangiopathic complications, especially neuropathy and arteriopathy, which are the basis for the development of foot ulcers leading to amputation, a major public health problem [1]. Therefore, there is a need to develop new methods for screening feet at risk, such as thermal imaging, which can detect hyperthermia in diabetic feet. The aim of this study is to evaluate the risk factors for developing foot ulcers and to assess the potential benefits of using a thermal camera.

## Materials and methods

Study design and ethical approval

This was a two-year prospective study conducted from February 2024 to February 2026. Ethical approval was obtained from the Ethics Committee (CEFZ/PR/06/02/24).

Study population

The study included diabetic patients admitted to the endocrinology and diabetology department of Agadir University Hospital.

Inclusion and exclusion criteria

All diabetic patients admitted during the study period, with or without foot ulcers, were included. Pregnant women and diabetic children were excluded.

Data collection and study variables

Data were collected using a standardized form. All parameters were extracted from hospital medical records and entered manually into an Excel spreadsheet (Microsoft Corporation, Redmond, WA, USA) while maintaining patient confidentiality. The variables collected included demographic data (age and gender) and diabetes-related characteristics (type of diabetes, duration since diagnosis in months, and history of smoking). Clinical evaluation of diabetic patients was also performed with systematic collection of clinical data.

Laboratory assessment included metabolic profiling with fasting blood glucose, HbA1c, and lipid profile. Glycemic control was defined as poorly controlled diabetes when HbA1c >8% and fasting blood glucose >2 g/L. Cardiovascular risk factors were assessed, including obesity (BMI >30 kg/m²) or overweight (BMI >25 kg/m²), hypertension (BP >130/80 mmHg), and hyperlipidemia (low-density lipoprotein cholesterol above the patient’s target).

Assessment of diabetic complications

Degenerative complications were systematically screened as follows: retinopathy detected by ophthalmic examination; kidney disease defined by microalbuminuria ≥30 mg/24 hours; kidney failure defined by creatinine clearance <60 mL/min; and neuropathy diagnosed when at least two of the following criteria were present: symptoms (pain and numbness), DN4 score >4/10, abnormal monofilament test, and absence of osteotendinous reflexes.

Peripheral arterial disease (PAD) was diagnosed in the presence of intermittent claudication and/or absent or weak pedal and posterior tibial pulses. A portable Doppler device was used to calculate the ankle-brachial index (ABI), defined as the ratio of systolic blood pressure at the ankle to that at the brachial artery. PAD was considered present when ABI <0.9 [1].

Diabetic foot risk classification

Risk classification was performed according to the International Working Group on the Diabetic Foot (IWGDF) system, which stratifies patients from low risk (Grade 0) to very high risk (Grade 3), based on the presence or absence of neuropathy, PAD, and history of foot ulcer or amputation [2].

Thermal imaging protocol

Thermal imaging was performed using a FLIR ONE Pro camera (Teledyne Technologies, Wilsonville, OR, USA) connected to a Samsung Galaxy S8 smartphone (Samsung Electronics Co., Ltd., Suwon, South Korea) and operated via an application developed by our team. The camera provides a thermal resolution of 160 × 120 pixels and operates in the 8-14 µm wavelength range, with a thermal accuracy of ±3 °C.

**Figure 1:**
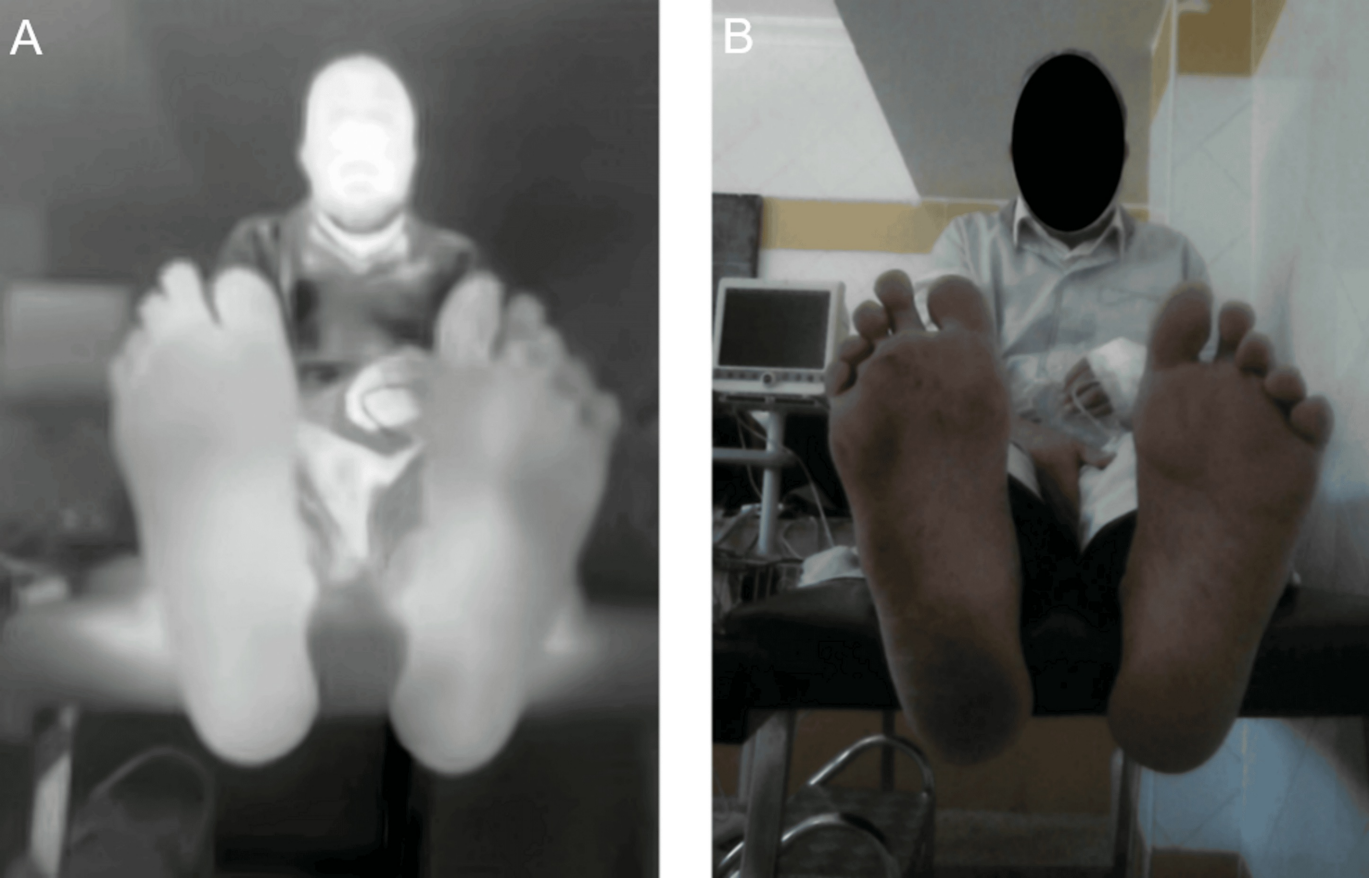
Example of acquisition: (A) thermal image and (B) RGB image

The system enables simultaneous acquisition of thermal infrared and standard visible light (RGB) images. These dual-image streams are spatially calibrated within the device, improving the accuracy of temperature mapping. An example of image acquisition is shown in Figure 1.

Acquisition protocol

**Figure 2:**
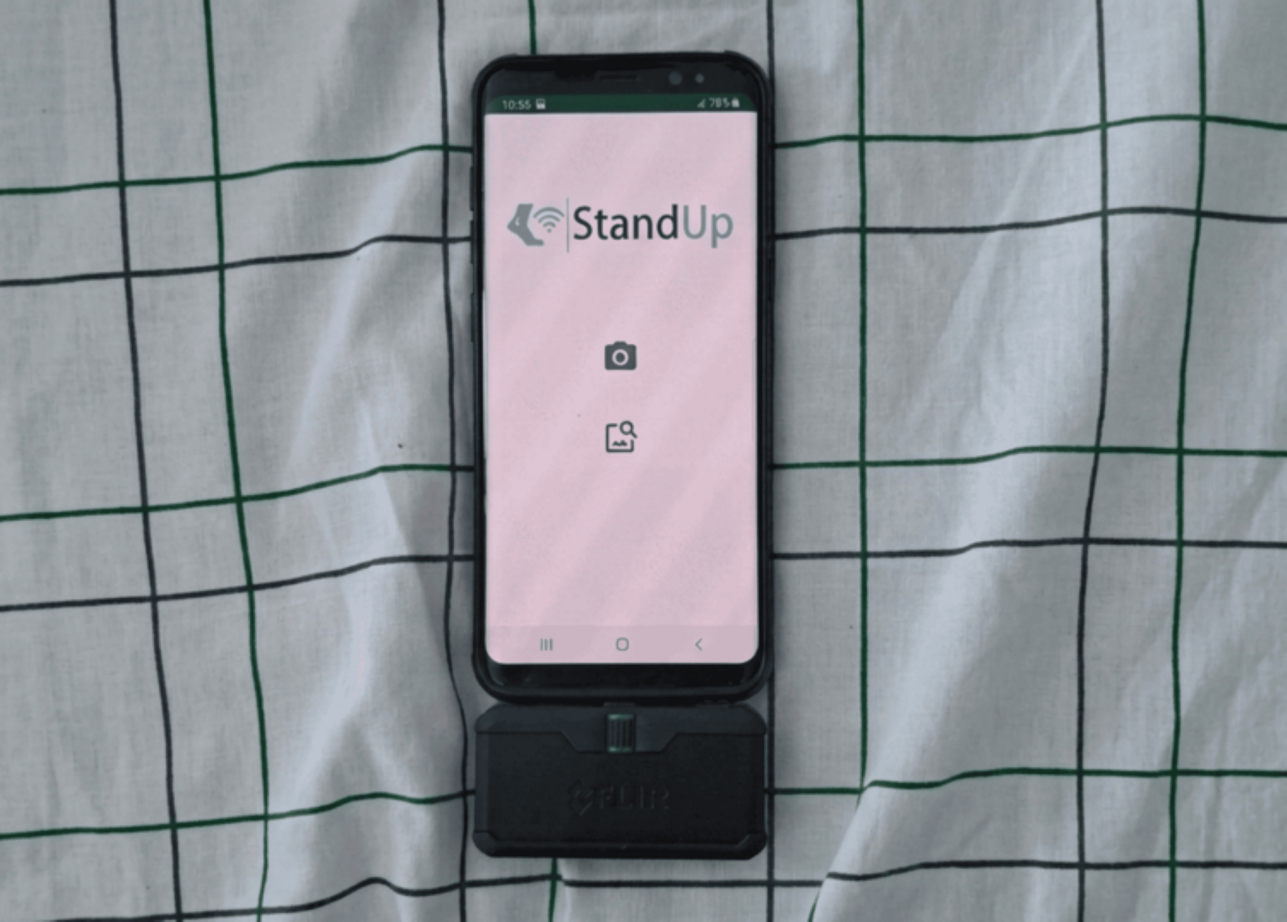
Acquisition using a FLIR ONE Pro camera interfaced with a Samsung Galaxy S8 smartphone, running an application (StandUp) developed by our team

Prior to the image acquisition procedure, each participant provided written informed consent and removed all footwear. A 15-minute acclimation period was then observed to ensure thermal stabilization of the feet. The participant was subsequently positioned lying on a stretcher, with the feet placed vertically at the edge and maintained at a fixed distance of 10 cm. A single freehand thermal image of the plantar feet was finally captured using a Samsung Galaxy S8 coupled with a FLIR ONE Pro infrared camera (Figure 2).

Segmentation of the plantar foot

Analysis of thermal images of the plantar foot requires precise and reliable delineation of the plantar regions corresponding solely to the two feet while excluding the background. This segmentation step is essential to ensure the correct extraction of thermal information and to avoid contamination by irrelevant areas. In this context, a convolutional neural network model based on a DE-ResUNet architecture [3] was adopted for the segmentation of thermal images. This model automatically and accurately isolates the plantar regions of both feet, relying on residual connections and an encoder-decoder structure optimized for the preservation of spatial details.

Registration of the plantar foot

**Figure 3:**
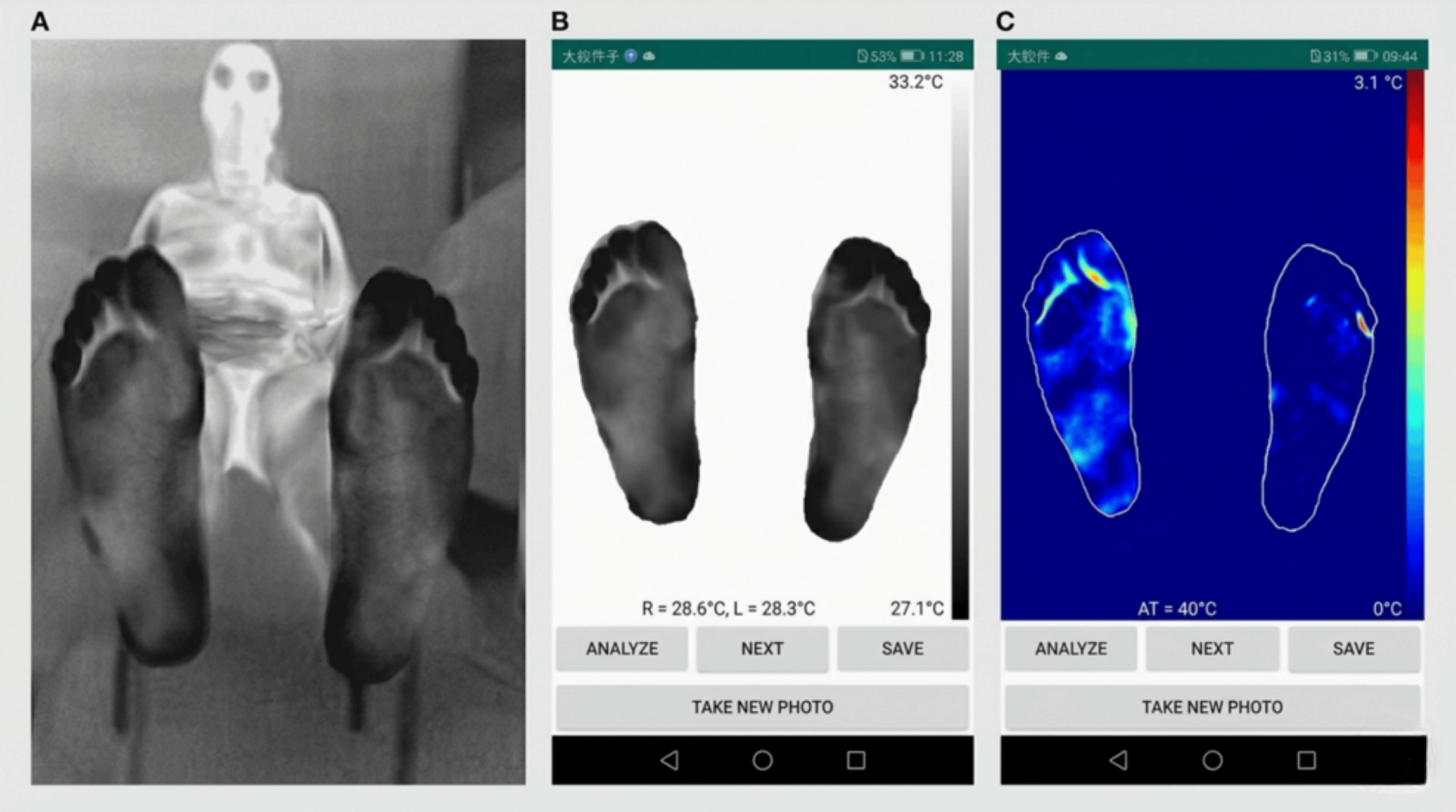
Segmentation of the plantar foot with calculation of the thermal delta between the right and left feet in a normal person

The computation of the absolute temperature difference requires prior alignment of the two contralateral feet, as they are not perfectly symmetrical mirror images. To achieve this, the thermal image was first divided into two regions corresponding to the right and left feet. The left foot image was then horizontally flipped to ensure a comparable orientation with the right foot. An Affine ConvNet model [4] was employed, demonstrating superior registration performance in terms of alignment accuracy and robustness. During the registration process, the right foot was considered the reference image, while the left foot was registered to it using the estimated affine transformation matrix (Figure 3). Hyperthermia with a high temperature difference was considered present when the delta was ≥2.2 °C [1].

Statistical analysis

Descriptive statistics were computed using IBM SPSS Statistics for Windows, version 31.0 (released 2025; IBM Corp., Armonk, NY, USA). An ANOVA test was performed to assess the significance of differences among the three ulceration risk groups. Pairwise comparisons, as well as comparisons between combined groups and the remaining group, were evaluated using independent-sample t-tests. For all analyses, the level of statistical significance was set at p < 0.05.

## Results

Data were collected from 158 diabetic patients during the study period who were admitted to the hospital and had thermal imaging performed. The results showed that 12.7% (n = 20) of these patients had hyperthermia with a significant difference in temperature between the right and left feet (delta >2.2), 65% (n = 13) of whom had a foot ulcer. Among patients with foot ulcers, 56% (n = 13/23) had a positive thermal imaging delta on admission, with a mean temperature difference of 3.3 ± 0.6 °C. The study population profile is detailed in Table 1.

**Table 1:** Characteristics of the sample of diabetic patients admitted to the department during the study period ABI, ankle-brachial index; IWGDF, International Working Group on the Diabetic Foot; LDL, low-density lipoprotein

Variable	N = 158
Age (years)	46.2 ± 18.6
Gender	
Female	68 (43.0%)
Male	90 (57.0%)
Time since diagnosis of diabetes (months)	47.9 ± 82.2
High blood pressure	
No	145 (91.8%)
Yes	13 (8.2%)
Smoking	
No	132 (83.5%)
Yes	26 (16.5%)
Overweight (BMI >25 kg/m²)	
No	146 (92.4%)
Yes	12 (7.6%)
Poor diabetes control	
No	103 (65.2%)
Yes	55 (34.8%)
High LDL cholesterol	
No	149 (94.3%)
Yes	9 (5.7%)
Kidney disease	
No	155 (98.1%)
Yes	3 (1.9%)
Diabetic retinopathy	
No	146 (92.4%)
Yes	12 (7.6%)
Diabetic neuropathy	
No	76 (48.1%)
Yes	82 (51.9%)
Peripheral artery disease (ABI < 0.9)	
No	112 (71.3%)
Yes	45 (28.7%)
Foot deformity	
No	135 (85.4%)
Yes	23 (14.6%)
Current foot ulcer	
No	135 (85.4%)
Yes	23 (14.6%)
Foot ulcer risk (IWGDF)	
Risk 0	74 (46.8%)
Risk 1	36 (22.8%)
Risk 2	25 (15.8%)
Risk 3	23 (14.6%)
Thermal delta >2.2 °C	
No	138 (87.3%)
Yes	20 (12.7%)

In our study, foot ulcer prevalence was 14.6% (n = 23). In patients with ulcers, the average age was 51 years, with a predominance of males (56%) (n = 13); the average time since diagnosis of diabetes was 6.5 years. High blood pressure was found in 26% (n = 6) of the patients, hyperlipidemia in 30% (n = 7), smoking in 47% (n = 11), and overweight in 34% (n = 8), with an average body mass index of 27 kg/m², poor diabetes control with an average hemoglobin A1c of 11%, and a high average fasting blood glucose level of 3.6 g/L.

**Figure 4:**
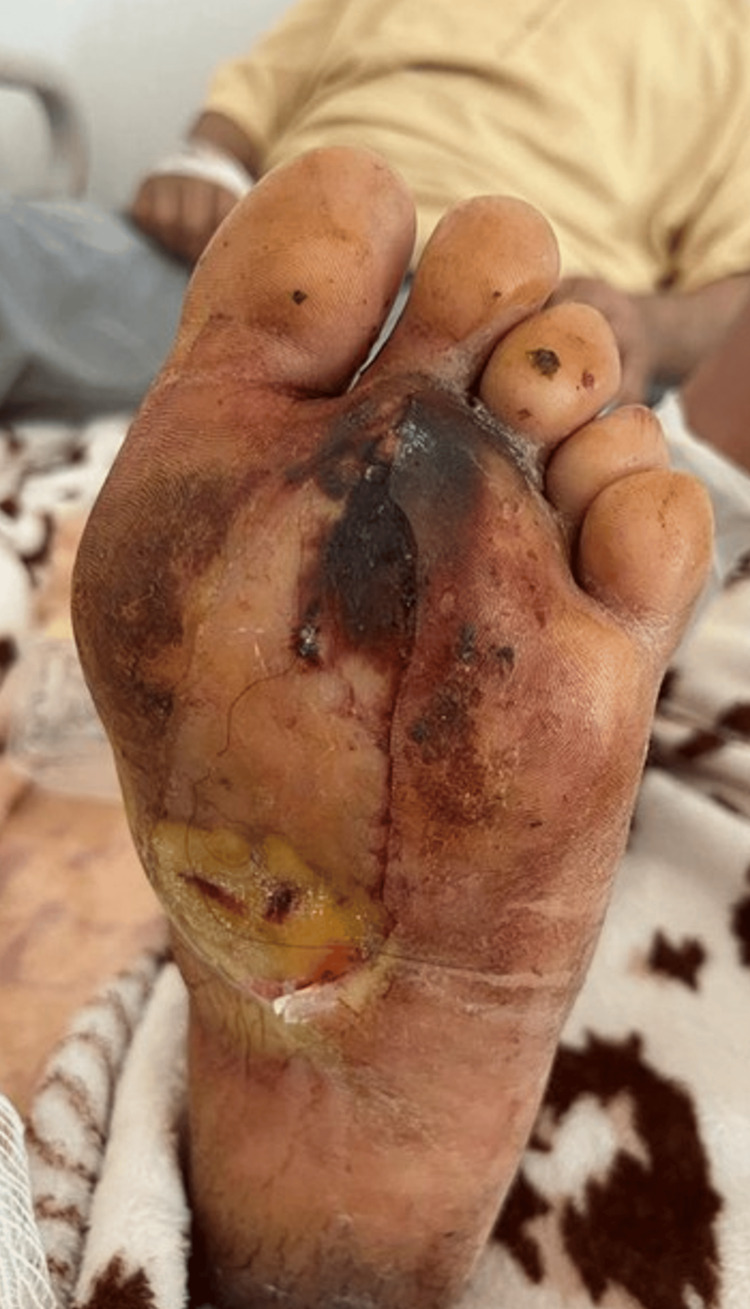
Photo of a patient with a foot ulcer

**Figure 5:**
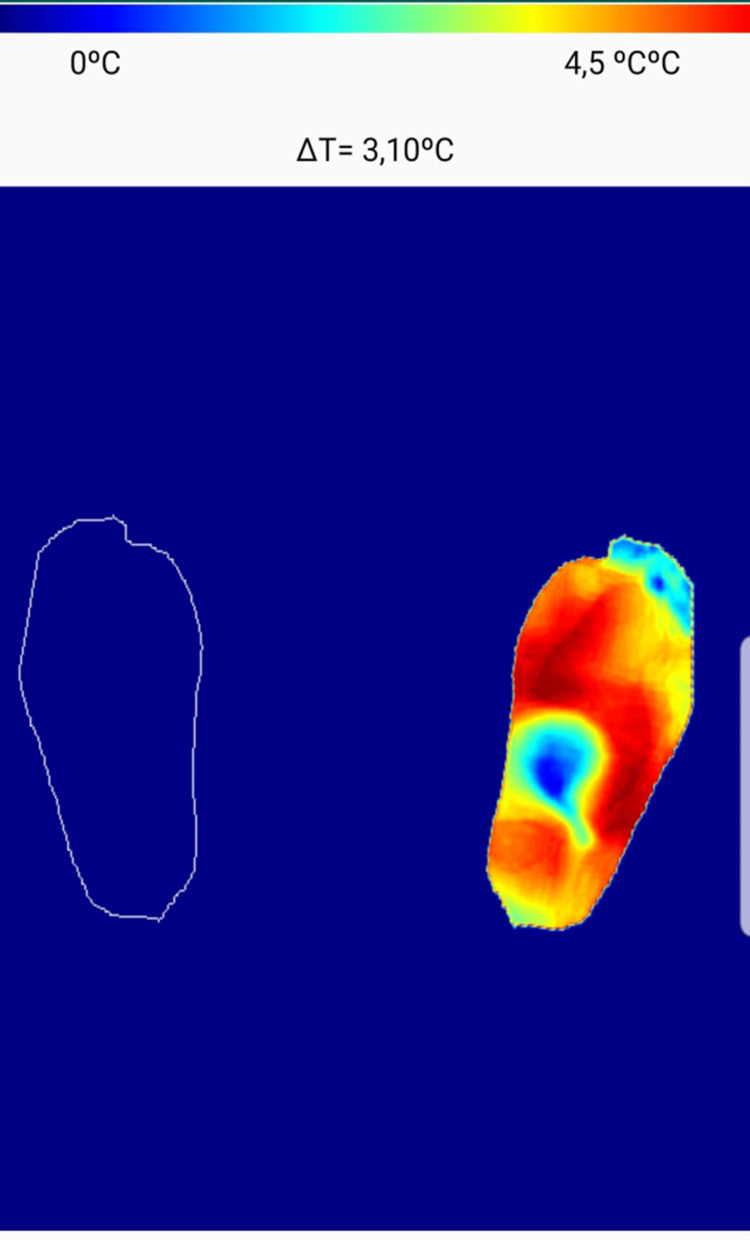
Thermal image of a patient with a foot ulcer and hyperthermia, with a high delta of 3.10 °C using an application on a smartphone developed by our team The red zone in the foot image indicates hyperthermia.

Foot examination of patients with ulcers revealed neuropathy in 86% (n = 20), peripheral artery disease with a low ABI in 82% (n = 19), and thermal imaging indicated hyperthermia and a high delta in 56% (n = 13/23) of patients with ulcers, with an average delta of 3.3 ± 0.6 °C (Figure 4, Figure 5).

A comparative analysis of risk factors among patients without foot ulcers (n = 135) and those with foot ulcers (n = 23) highlighted several factors associated with the presence of an ulcer (Table 2). Smoking (p = 0.001), overweight (p = 0.017), and poor glycemic control (p = 0.002) were all significantly more common in the group with ulcers. Degenerative complications were also statistically more common: diabetic retinopathy (0% vs. 52.2%, p < 0.001), diabetic neuropathy (45.9% vs. 87.0%, p < 0.001), peripheral artery disease (19.4% vs. 82.6%, p < 0.001), and foot deformity (5.9% vs. 65.2%, p < 0.001). Regarding foot risk, all patients without ulcers were classified as risk 0, 1, or 2, and all patients with ulcers were classified as risk 3 (p < 0.001). Furthermore, a high thermal delta on thermal imaging was significantly more common in the group with ulcers (56.5% vs. 5.2%, p < 0.001) (Table 2).

**Table 2:** Comparison of risk factors between patients without foot ulcers (n = 135) and those with foot ulcers (n = 23) Significant p-values were defined as p < 0.05. ABI, ankle-brachial index; IWGDF, International Working Group on the Diabetic Foot; LDL, low-density lipoprotein

Variable	No ulcer (n = 135)	With ulcer (n = 23)	p-Value
Age (years)	45.4 ± 18.8	50.7 ± 17.0	0.211
Time since diagnosis of diabetes (months)	42.9 ± 79.6	77.7 ± 92.5	0.06
Gender	74 (54.8%)	16 (69.6%)	0.055
High blood pressure	9 (6.7%)	4 (17.4%)	0.099
Smoking	16 (11.9%)	10 (43.5%)	0.001
Overweight (BMI >25 kg/m²)	7 (5.2%)	5 (21.7%)	0.017
High blood sugar	40 (29.6%)	15 (65.2%)	0.002
High LDL cholesterol	7 (5.2%)	2 (8.7%)	0.620
Kidney disease	1 (0.7%)	2 (8.7%)	0.056
Diabetic retinopathy	0 (0%)	12 (52.2%)	0.001
Diabetic neuropathy	62 (45.9%)	20 (87%)	0.001
Peripheral artery disease (ABI < 0.9)	26 (19.4%)	19 (82.6%)	0.001
Foot deformity	8 (5.9%)	15 (65.2%)	0.001
Foot ulcer risk (IWGDF)			
Risk 0	74 (54.8%)	0 (0%)	0.001
Risk 1	36 (26.7%)	0 (0%)	0.002
Risk 2	25 (18.5%)	0 (0%)	0.026
Risk 3	0 (0%)	23 (100%)	0.001
Thermal delta >2.2 °C	7 (5.2%)	13 (56.5%)	0.001

We compared patients with a high temperature delta on thermal imaging with risk factors for foot ulcers and found a significant positive correlation with time since diagnosis of diabetes (p = 0.001), smoking (p < 0.001), high blood pressure (p = 0.003), overweight (p < 0.001), high blood sugar (p < 0.001), hyperlipidemia (p = 0.003), retinopathy (p < 0.001), kidney disease (p < 0.001), neuropathy (p < 0.001), PAD with ABI < 0.9 (p < 0.001), foot malformation (p < 0.001), and foot ulcer risk (p < 0.001).

The study found that 5% (n = 7) of patients without a foot ulcer had a high delta on thermal imaging. The two groups of patients, those with a delta >2.2 with or without a foot ulcer, were compared with regard to foot risk factors. We found a statistically significant correlation in patients with a high delta temperature and no foot ulcer with retinopathy (p = 0.001), foot ulcer risk (p = 0.001), and thermal delta (p = 0.001), with an average delta of 2.4 ± 0.2 °C in feet without ulcers and an average of 3.3 ± 0.6 °C in feet with ulcers (Table 3).

**Table 3:** Comparison of the two groups with a delta >2.2 °C with or without a foot ulcer Significant p-values were defined as p < 0.05. ABI, ankle-brachial index; IWGDF, International Working Group on the Diabetic Foot; LDL, low-density lipoprotein

Variable	Delta ≥ 2.2 °C without ulcer (n = 7)	Delta ≥ 2.2 °C with ulcer (n = 13)	p-Value
Age (years)	52.0 ± 16.4	51 ± 20.3	0.926
Time since diagnosis of diabetes (months)	77.1 ± 89.8	116.3 ± 101.8	0.405
Male gender	3 (43%)	9 (69%)	0.503
High blood pressure	1 (14%)	4 (31%)	0.787
Smoking	2 (29%)	8 (62%)	0.348
Overweight (BMI >25 kg/m²)	2 (29%)	5 (38%)	1.000
High blood sugar	6 (86%)	12 (92%)	1.000
High LDL cholesterol	2 (29%)	2 (15%)	0.907
Kidney disease	1 (14%)	2 (15%)	1.000
Diabetic retinopathy	0 (0%)	12 (92%)	0.001
Diabetic neuropathy	7 (100%)	11 (85%)	0.755
Peripheral artery disease (ABI < 0.9)	6 (86%)	13 (100%)	0.747
Foot deformity	2 (29%)	7 (54%)	0.540
Foot ulcer risk (IWGDF)			
Risk 1	2 (29%)	0 (0%)	0.001
Risk 2	5 (71%)	0 (0%)	0.001
Risk 3	0 (0%)	13 (100%)	0.001
Thermal delta (°C)	2.4 ± 0.2	3.3 ± 0.6	0.001

## Discussion

The global incidence of diabetic foot ulcers is between 3% and 10% [2]. The prevalence of foot ulcers in Morocco is unknown; this is the first study conducted in southern Morocco to determine this prevalence. In our study, the prevalence was 14.6%, which is comparable to that observed in other African studies [5,6]. Among diabetic patients with ulcers, the mean age was 50 years. The mean age reported in the European literature is 68.94 years [7]. The predominance of males is a phenomenon confirmed by several authors. Sani et al. [8] found a sex ratio of 2.46. The average time since diagnosis was 8.5 years (range: 0-23 years). Diabetic foot ulcer revealed undiagnosed diabetes in seven patients. These findings are confirmed by various Moroccan studies [9,10].

Foot ulcer risk factors

In our study, the main cardiovascular risk factors associated with foot ulcers were high blood pressure in 17% of patients, hyperlipidemia in 84%, smoking in 44%, and overweight in 22%, which are primary risk factors for atherosclerosis identified in the published research [11]. Xia et al. presented a study confirming the role of smoking in all stages of foot ulcers, from development to healing [12].

Based on published studies, poor diabetes control and the presence of diabetic complications (retinopathy, neuropathy, and peripheral artery disease) increase the risk of foot ulcers [13]. In our series, we found a significant correlation between all of these factors and the development of a foot ulcer (p < 0.05).

Contribution of thermal imaging to the diabetic foot

Thermal imaging is a noninvasive, non-irradiating imaging technique that measures and maps skin temperature distribution in real time. When applied to the diabetic foot, it is based on the principle that inflammation, neuropathy, and ischemia cause local changes in skin perfusion, resulting in thermal asymmetries between the two feet. Armstrong et al. [14] laid the scientific foundation for the use of thermal imaging in high-risk diabetic feet. They showed that comparative measurement of skin temperature between the two feet could detect clinical abnormalities even before the development of a foot ulcer.

Plantar hyperthermia is defined as a temperature difference greater than 2.2 °C between an area of the foot and the symmetrical area of the contralateral foot; this temperature difference has become the international standard for the early detection of ulcer risk. In a follow-up randomized clinical trial, Armstrong et al. [15] confirmed the early predictive value of this thermal indicator: patients who developed an ulcer had a temperature differential 4.8 times higher at the ulceration site in the week preceding the onset of the ulcer compared to non-ulcerated patients (3.50 ± 1.0 °C vs. 0.74 ± 0.05 °C, p = 0.001). These results highlight the significant value of the thermal camera as a tool for the early detection of diabetic foot at risk. In our population, 5% of patients without ulcers were found to have hyperthermia with an average temperature difference of 2.4 ± 0.2 °C.

Thermal imaging and peripheral neuropathy

**Figure 6:**
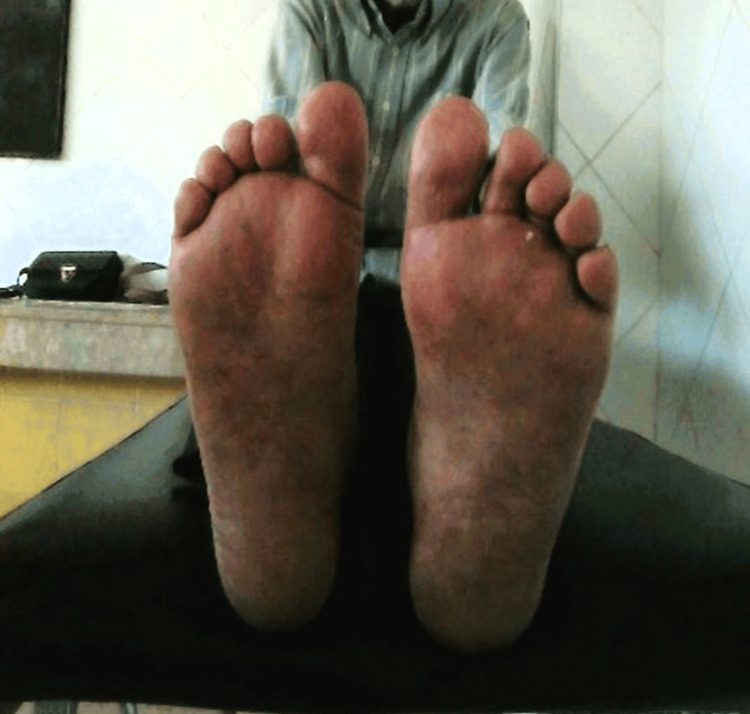
Image of a patient with neuropathy (left foot DN4 score of 7/10) without an ulcer

**Figure 7:**
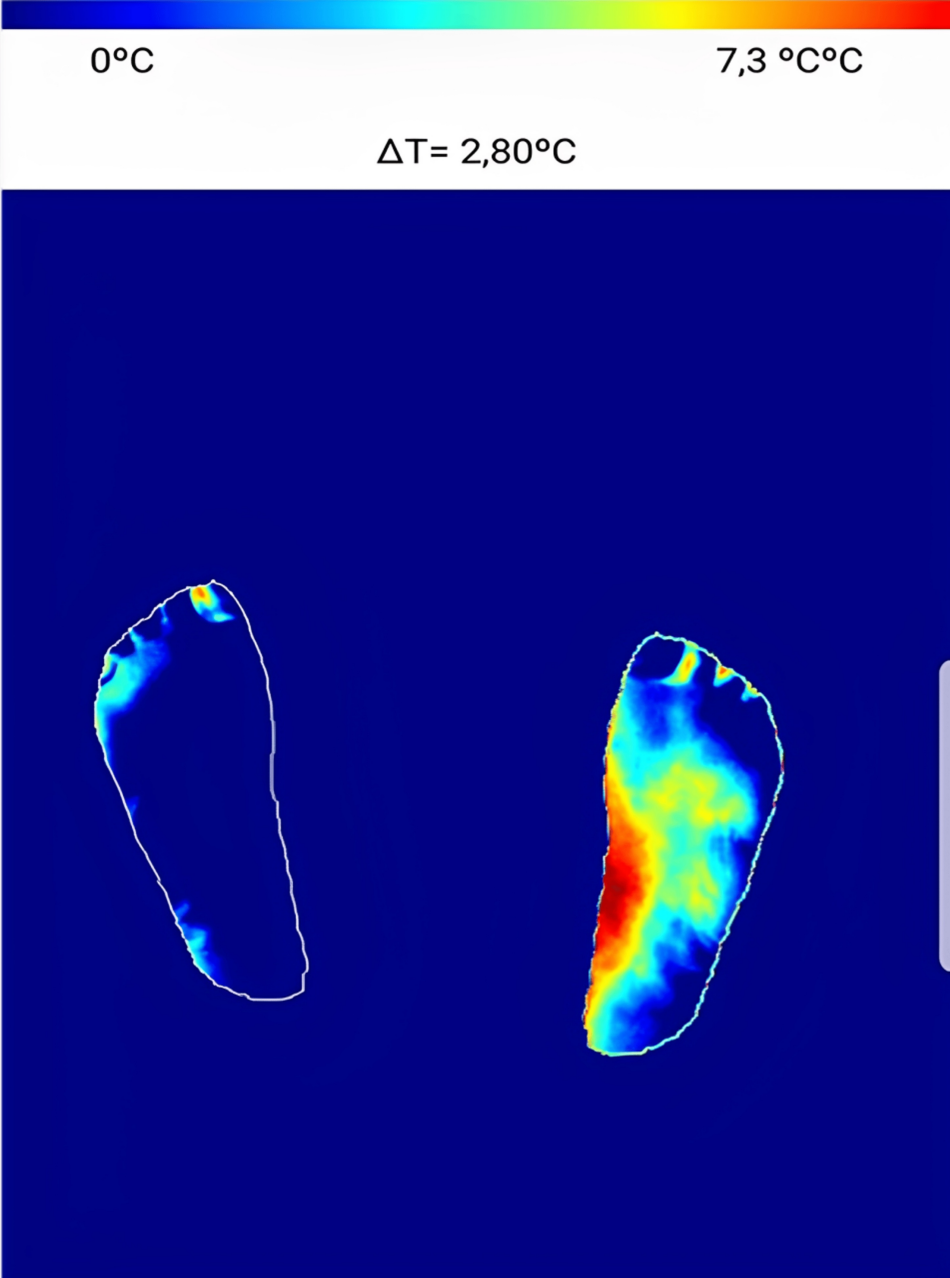
Thermal image of a patient with neuropathy without ulcers showing hyperthermia in the left foot with a positive temperature difference of 2.8 °C using an application on a smartphone developed by our team The red zone in the foot image indicates hyperthermia.

Diabetic peripheral neuropathy is one of the most common complications of diabetes, but it can be difficult to detect early. Thermal imaging has shown its ability to detect the slight temperature variations associated with this disorder. By measuring the heat emitted by the skin, it can reveal early warning signs of neuropathy even before noticeable symptoms appear, allowing for early intervention. Diabetic patients with neuropathy have higher temperatures (between 32 and 35 °C) compared to those without neuropathy (27-30 °C), and hyperthermia of the foot is observed with a temperature difference between the two feet greater than 2.2 °C (Figure 6, Figure 7) [16].

Foot neuropathy is identified in studies as a major risk factor for foot ulcers. In our study, the patient group with a positive temperature difference showed a positive correlation with the presence of neuropathy.

Thermal imaging and PAD

Thermal imaging can be used to assess blood perfusion by measuring skin temperature. Variations in skin temperature may indicate abnormal blood circulation; PAD can lead to reduced blood flow to the foot and a colder foot. In studies, the cutoff temperature value for foot hypothermia has not yet been established.

Peripheral artery disease of the foot has been identified in studies as a major risk factor for foot ulcers. Reduced blood flow delays ulcer healing and increases the risk of infection and necrosis [17]. In our study, within the group with ulcers, we found a positive correlation with peripheral artery disease with a low ABI.

Thermal imaging and foot ulcers

Foot ulcers in diabetic patients are a serious and common complication. Thermal imaging can be used to monitor these ulcers by detecting changes in temperature within the affected area. An increase in temperature may indicate infection or inflammation, which may require immediate medical intervention, while a decrease in temperature of the ulcerated foot may indicate a reduction in inflammation and the start of healing. In our study, we found that 56% (n = 13/23) of patients with ulcers had a positive thermal imaging delta on admission, with an average delta of 3.3 ± 0.6 °C.

In patients with a positive delta on thermal imaging without an ulcer, a significant correlation was found with retinopathy, the first microvascular complication to appear in diabetics, which can be considered a marker of cardiovascular risk [18].

A positive correlation was also found between the group presenting a positive delta on thermal imaging without ulcers and the risk of foot ulcers, which supports studies that have shown an increased foot temperature (delta >2.2) appears 15 days before the development of an ulcer, potentially serving as a predictive factor for ulcer development [15,19].

Thermal imaging and telemedicine

Thermal imaging is a noninvasive, fast, and low-cost technique that can be used to identify diabetic patients at the highest risk of developing an ulcer [20], which patients themselves can use to regularly monitor their feet, allowing healthcare providers to detect foot ulcers earlier and initiate appropriate treatments to prevent amputations. The IWGDF recommends regular foot monitoring in patients at risk and recognizes the potential of new technologies, including telemedicine and plantar temperature monitoring, for early detection of foot complications and prevention of ulceration [1].

Limitations

This study is limited by its single-center design and relatively small sample size. The hospital-based population may introduce selection bias, and the absence of longitudinal follow-up limits the evaluation of predictive outcomes of thermal imaging. Our study was conducted at a Level 3 university hospital and included patients with complicated and severely uncontrolled diabetes, which explains the high prevalence of diabetic foot ulcers in our sample.

## Conclusions

Diabetic foot remains a major public health issue. Assessing the risk of foot ulcers requires time, trained medical staff, and expensive vascular testing. This is why it is important to develop new, faster, and noninvasive technologies that can detect at-risk feet, such as thermal imaging cameras, which can detect hyperthermia 15 days before an ulcer appears, enabling earlier treatment to prevent amputations. Our future goal is to develop an application that can also detect hypothermia in cases of reduced blood flow due to peripheral artery disease, using thermal image segmentation of the foot based on vascular regions corresponding to the affected artery.
